# Systems-level identification of conserved molecular drivers underlying the progression of alcoholic hepatitis and alcoholic cirrhosis and their therapeutic modulation by S-adenosyl-L-methionine

**DOI:** 10.3389/fbinf.2026.1892336

**Published:** 2026-07-15

**Authors:** Prasanth Babu Nandagopal, Gayatri Munieswaran, Venkatraman Manickam

**Affiliations:** School of Biosciences and Technology, Vellore Institute of Technology (VIT), Vellore, Tamil Nadu, India

**Keywords:** alcoholic cirrhosis, alcoholic hepatitis, hub genes, molecular dynamics, protein-protein interaction, SAMe, TGFB1

## Abstract

**Introduction:**

Alcohol-associated liver disease (ALD) encompasses a progressive spectrum of hepatic injury, with alcoholic hepatitis (AH) and alcoholic cirrhosis (AC) representing clinically severe and mechanistically interconnected stages. Despite significant disease burden, therapeutic strategies targeting core molecular drivers of disease progression remain limited. Identifying conserved regulatory determinants across AH and AC may provide a rational framework for mechanism-driven therapeutic intervention. S-adenosyl-L-methionine (SAMe), a key metabolic intermediate involved in methylation and redox homeostasis, has shown hepatoprotective potential; however, its direct molecular targets in ALD remain poorly characterized.

**Methodology:**

An integrative *in silico* framework was employed to identify conserved molecular signatures and evaluate SAMe-target interactions. Publicly available transcriptomic datasets from the NCBI Gene Expression Omnibus (GEO) were analysed to identify differentially expressed genes (DEGs) in AH and AC, followed by Venn-based intersection to determine shared DEGs. Functional enrichment (GO and KEGG) and protein-protein interaction (PPI) network analyses were conducted to identify key regulatory hub genes. Selected hub proteins were subjected to molecular docking with SAMe and the stability of the resulting protein-ligand complexes was further evaluated using 100ns molecular dynamics (MD) simulations in conjunction with MM/GBSA binding free energy calculations.

**Results:**

This integrative analysis identified 826 shared DEGs enriched in pathways associated with intracellular signalling, transcriptional regulation and extracellular matrix (ECM) organization. Network analysis revealed TGFB1, COL1A2, ESR1, PDGFRA, LUM and BCL2 as central hub genes. Molecular docking demonstrated favourable binding interactions of SAMe with these targets, with TGFB1 exhibiting the highest binding affinity (−7.0 Kcal/mol). MD simulations confirmed stable conformational dynamics of SAMe-bound complexes, particularly TGFB1, characterized by reduced structural fluctuations, increased compactness and sustained hydrogen bonding. Binding free energy analysis further supported the thermodynamic stability of these interactions, with the TGFB1-SAMe complex showing the most favourable energy profile.

**Discussion:**

Collectively, these findings identify conserved molecular signatures linking AH and AC and suggest potential molecular interactions between SAMe and key regulatory proteins implicated in disease progression. By integrating transcriptomic, network and structural analyses, this study provides a systems-level framework for understanding the molecular landscape of ALD and offers a basis for future experimental studies aimed at evaluating therapeutic strategies targeting shared disease determinants.

## 1 Introduction

Alcohol-associated liver disease (ALD) represents a major global health burden and remains a leading cause of chronic liver-related morbidity and mortality worldwide (Narro et al., 2024). Harmful alcohol consumption accounts for approximately 2.6 - 3 million deaths annually, representing nearly 5% of overall deaths worldwide, with a substantial proportion attributed to alcohol-associated liver complications (World Health Organization, 2024). Given its central role in metabolic homeostasis, the liver is the primary site of ethanol metabolism and is therefore particularly vulnerable to alcohol-induced injury (Wilson and Matschinsky, 2020). Ethanol is predominantly oxidized via alcohol dehydrogenase and the microsomal ethanol-oxidizing system, leading to the generation of reactive metabolites such as acetaldehyde and reactive oxygen species (Wu and Cederbaum, 2003). These metabolites disrupt cellular redox balance, impair mitochondrial function, promote lipid accumulation and inflammatory signalling, thereby creating a pathogenic environment conducive to liver injury (Hoek et al., 2002; Yan et al., 2023; Lee et al., 2025). Consequently, excessive and sustained alcohol exposure initiates a spectrum of hepatic damage that includes hepatic steatosis, characterized by abnormal lipid accumulation in hepatocytes and alcoholic hepatitis, a severe inflammatory condition marked by hepatocellular damage and immune activation (Osna et al., 2017; Ha et al., 2022). Persistent alcohol exposure further promotes fibrogenesis and progressive hepatic fibrosis, ultimately leading to cirrhosis, an advanced stage defined by extensive scar formation, architectural distortion of the liver parenchyma and compromised hepatic function (Mackowiak et al., 2024; Mandrekar and Mandal, 2024). In certain cases, long-standing cirrhotic injury can predispose individuals to the development of hepatocellular carcinoma (HCC), further complicating disease outcomes (Morgan et al., 2004; Huang et al., 2022). Among the clinical manifestations of ALD, alcoholic hepatitis (AH) and alcoholic cirrhosis (AC) represent particularly critical disease states due to their association with high short- and long-term mortality, thereby imposing substantial clinical and socioeconomic burdens. Although clinically distinct, these conditions are mechanistically interconnected and share overlapping pathophysiological processes, including chronic inflammation, immune dysregulation, oxidative stress and extracellular matrix remodelling. These shared mechanisms are central to disease initiation, progression and severity across the ALD spectrum.

Despite the substantial clinical burden of ALD, therapeutic options for AH and AC remain limited and largely supportive (Kong et al., 2019; Arab et al., 2023). Corticosteroids are currently the most commonly employed pharmacological intervention for severe AH. However, their clinical benefit is modest, restricted to a subset of patients and often accompanied by significant adverse effects, including increased susceptibility to infections and gastrointestinal complications (Thursz and Morgan, 2016). For patients with advanced cirrhosis, liver transplantation represents the only definitive treatment, yet its applicability is constrained by stringent eligibility criteria, limited donor availability and ethical considerations (Singal et al., 2013; Grąt et al., 2014; Lucey, 2014). In addition, other pharmacological agents, including antioxidants, anti-inflammatory therapies and biologics, have been widely investigated; however, their efficacy remains inconsistent. Notably, no FDA-approved molecularly targeted therapies currently exist that directly address the underlying pathogenic mechanisms driving ALD progression. This therapeutic gap reflects an incomplete understanding of the molecular determinants governing disease onset and inter-stage transition, highlighting a critical need for mechanism-based, target-driven therapeutic strategies. Addressing this unmet need requires the identification of shared molecular determinants underlying AH and AC, as these are likely to represent core regulators of disease biology rather than stage-specific effects. Targeting such conserved pathways offers the potential to modulate disease progression across multiple stages of ALD and to identify therapeutic vulnerabilities with broader translational relevance.

In this context, targeting key metabolic pathways that regulate redox balance and cellular homeostasis has emerged as a promising strategy for developing mechanism-based therapeutic interventions in ALD. One such pathway that has received increasing attention in the context of liver injury is the methionine cycle, which plays a pivotal role in maintaining cellular methylation capacity and redox homeostasis (Li et al., 2020). A central intermediate in this pathway is S-adenosyl-L-methionine (SAMe), a principal methyl donor involved in transmethylation reactions, epigenetic regulation, polyamine synthesis and phospholipid metabolism (Lu, 2000). In hepatic tissue, SAMe contributes to antioxidant defence by supporting glutathione synthesis and maintaining redox balance, thereby exerting hepatoprotective effects under conditions of oxidative and inflammatory stress. Consistent with these mechanistic roles, SAMe has been extensively investigated for its therapeutic potential in mitigating alcohol-induced oxidative and metabolic liver injury (Lieber, 2002a). Multiple experimental and clinical studies have demonstrated that SAMe supplementation can enhance hepatic glutathione levels and attenuate liver injury in alcohol-related liver diseases (Lieber et al., 1990; Cederbaum, 2010). In line with these findings, our recent co-supplementation study involving SAMe and B-vitamins demonstrated significant hepato-pancreatic protection in alcohol-induced injury in C57BL/6 J mice (Nandagopal et al., 2026b). Furthermore, in our zebrafish study, SAMe exhibited the potential to reverse ethanol-induced developmental toxicity, primarily through restoration of glutathione homeostasis and modulation of oxidative stress pathways (Nandagopal et al., 2026a). Despite accumulating experimental and clinical evidence supporting the hepatoprotective potential of SAMe, the precise molecular mechanisms and specific mediators underlying its therapeutic effects remain incompletely defined. In this context, evaluating the potential of SAMe to modulate shared molecular determinants underlying both AH and AC represents a biologically and clinically relevant strategy, given the convergence of oxidative, inflammatory and fibrotic pathways across these disease states. Systematic characterization of SAMe-target interactions within these shared molecular networks may enable the identification of key regulatory nodes through which SAMe exerts its effects. Such a network-oriented approach not only facilitates the delineation of the molecular mechanisms underlying SAMe-mediated hepatoprotection but also supports the selective targeting of critical molecular drivers linking AH and AC.

Given the complexity and heterogeneity of ALD, integrative computational approaches offer a robust framework for identifying conserved molecular determinants across related disease states (Zanello et al., 2023). Despite advances in transcriptomic and network-based analyses in liver disease, a systematic cross-condition analysis integrating AH and AC to delineate shared molecular drivers and evaluate their therapeutic modulation remains lacking. To address this gap, the present study employs an integrative systems biology framework to identify conserved molecular signatures underlying AH and AC. Transcriptomic datasets from both conditions were systematically integrated to identify shared differentially expressed genes, followed by functional enrichment and protein-protein interaction network analyses to prioritize key hub genes with potential regulatory significance. Importantly, this study extends beyond conventional transcriptomic profiling by incorporating structure-based approaches to evaluate the therapeutic relevance of identified targets. These candidate genes were subsequently assessed for their interaction with SAMe using molecular docking and the stability of the resulting complexes was further examined through molecular dynamics simulations. By combining cross-condition transcriptomic integration, network-based target prioritization and molecular-level validation, this work provides a novel systems-level framework to elucidate shared pathogenic mechanisms and to explore the mechanistic basis of SAMe-mediated modulation. Collectively, this approach establishes a rational foundation for the identification of network-informed therapeutic targets and supports the development of mechanism-driven strategies to mitigate disease progression across the ALD spectrum.

## 2 Methods

### 2.1 Screening of hub genes

#### 2.1.1 Data collection, curation and differential gene expression analysis

Gene expression profiles associated with alcoholic hepatitis (AH) and alcoholic cirrhosis (AC) were obtained from the NCBI Gene Expression Omnibus (GEO) (https://www.ncbi.nlm.nih.gov/geo/), a publicly accessible repository of high-throughput transcriptomic datasets (Barrett et al., 2005). Following a comprehensive evaluation of publicly available datasets, GSE142530 was identified as the most suitable cohort meeting the study objectives and inclusion criteria, with sufficient sample representation to facilitate the identification of shared molecular signatures associated with disease progression. The dataset GSE142530 was selected due to its relevance to ALD and its inclusion of high-throughput transcriptomic profiles from human liver samples encompassing AH, AC and appropriate control groups. Differential gene expression analysis was performed using the GEO2R tool, which implements the DESeq2 package for RNA-seq datasets. Pairwise comparisons were conducted between control and alcoholic hepatitis (C vs. AH) samples and between control and alcoholic cirrhosis (C vs. AC) samples to identify differentially expressed genes (DEGs) associated with each disease state (Love et al., 2014). Genes with an adjusted *p*-value <0.05 and an absolute log_2_ fold change (|log_2_FC|) > 1 were considered significantly differentially expressed. To account for multiple hypothesis testing, adjusted *p*-values were calculated using the Benjamini–Hochberg false discovery rate (FDR) correction method (Benjamini and Hochberg, 1995). Volcano plots were subsequently generated to visualize the distribution of DEGs. Shared DEGs between AH and AC were identified through intersection analysis using Venny 2.1.0 and the resulting common gene set was subsequently selected for downstream functional enrichment and network-based analyses (https://bioinfogp.cnb.csic.es/tools/venny/) (Oliveros, 2026).

#### 2.1.2 Functional enrichment analysis

To elucidate the biological significance of the shared DEGs identified between AH and AC, functional enrichment analysis was performed using FunRich (http://www.funrich.org/) (Pathan et al., 2015). Prior to analysis, duplicate entries were removed and gene identifiers were standardized to official gene symbols to ensure consistency. Gene Ontology (GO) enrichment analysis was conducted to classify DEGs into three principal categories: biological process (BP), molecular functions (MF) and cellular components (CC). Enrichment significance was determined by comparing the proportion of input genes associated with specific GO terms against a background reference dataset (*Homo sapiens*) using the hypergeometric test. GO terms with an adjusted *p*-value (FDR) < 0.05 were considered significantly enriched. To further characterize pathway-level alterations, Kyoto Encyclopedia of Genes and Genomes (KEGG) pathway enrichment analysis was performed using the Kyoto Encyclopedia of Genes and Genomes (https://www.genome.jp/kegg/pathway.html) (Kanehisa and Goto, 2000). DEGs were mapped onto curated signalling and metabolic pathways to identify overrepresented functional modules associated with disease progression. Pathways with an adjusted *p*-value (FDR) < 0.05 were considered significantly enriched.

#### 2.1.3 Protein-protein interaction network analysis and hub gene identification

To investigate the interaction landscape of the shared DEGs, protein-protein interaction (PPI) network analysis was performed using the STRING database (version 12.0) (https://string-db.org/) (Szklarczyk et al., 2023). The list of significant DEGs (*p* < 0.05) was submitted to STRING with *H. sapiens* selected as the reference organism. Both experimentally validated and predicted interactions, including co-expression evidence, were considered for network construction. A minimum interaction confidence score of 0.4 was applied, corresponding to medium-confidence interactions, while scores of 0.7–0.9 and 0.9–0.99 represented high- and highest-confidence interactions, respectively. The resulting PPI network was imported into Cytoscape (version 3.10.4) for visualization and further topological analysis (https://cytoscape.org/) (Shannon et al., 2003). Network refinement was performed by removing duplicate edges and isolated nodes to enhance analytical robustness. Key topological parameters, including degree centrality (number of direct interactions), betweenness centrality (number of shortest paths passing through a node) and closeness centrality (inverse of the average shortest path length to all other nodes), were computed using the built-in NetworkAnalyzer tool. Hub gene identification was performed using the CytoHubba plugin (Chin et al., 2014). To improve prioritization robustness, the top ten genes ranked by each centrality metric were compared and genes common to all three rankings were considered robust hub candidates. These candidates were further prioritized based on the magnitude of differential expression, functional enrichment profiles and supporting literature evidence and were subsequently selected for downstream structural and interaction analyses.

### 2.2 Analysis of binding affinity and molecular dynamics

To evaluate the therapeutic potential of selected targets in ALD, structure-based computational analyses were performed to assess binding affinity and molecular behaviour. S-adenosyl-L-methionine (SAMe), a biologically active methyl donor with established roles in hepatic metabolism and redox regulation, was selected as the candidate ligand for interaction studies. Based on PPI network analysis and functional pathway prioritization, six core hub proteins were selected as targets for downstream structural investigation: transforming growth factor beta 1 (TGFB1), collagen type I alpha 2 chain (COL1A2), estrogen receptor 1 (ESR1), lumican (LUM), platelet-derived growth factor receptor alpha (PDGFRA) and B-cell lymphoma-2 (BCL2). These proteins represent key regulatory nodes implicated in inflammation, extracellular matrix remodelling, fibrogenesis and cell survival pathways associated with the progression of AH and AC. Subsequent analyses were designed to characterize the binding interactions between SAMe and these target proteins, as well as to evaluate the structural stability and dynamic behaviour of the resulting complexes.

#### 2.2.1 Homology modelling and structural validation of target proteins

For proteins lacking experimentally resolved structures, namely, COL1A2 and LUM, homology modelling was performed using the SWISS-MODEL web server (https://swissmodel.expasy.org/) (Schwede et al., 2003; Waterhouse et al., 2018). FASTA sequences retrieved from UniProt (COL1A2 - P08123, LUM - P51884) were used as input, with template selection based on sequence identity and E-value thresholds (<1 × 10^−5^) (Bairoch et al., 2004; Bateman et al., 2017). Homology models were evaluated based on Global Model Quality Estimation (GMQE) scores and sequence identity, where models with GMQE values approaching 1 and sequence identity greater than 50% were considered to structural reliable. The predicted structures were subsequently subjected to comprehensive structural validation using PROCHECK, ERRAT, VERIFY3D and ProSA. Ramachandran plot analysis generated through PROCHECK was used to evaluate stereochemical quality by assessing backbone dihedral angles and residue geometry (https://www.ebi.ac.uk/thornton-srv/software/PROCHECK/) (Laskowski et al., 2012). ERRAT was employed to analyse non-bonded atomic interactions and compute an overall quality factor (https://www.doe-mbi.ucla.edu/errat/) (Colovos and Yeates, 1993; Dym et al., 2006), while VERIFY3D assessed the compatibility between the three-dimensional structures and their corresponding amino acid sequences (https://www.doe-mbi.ucla.edu/verify3d/) (Bowie et al., 1991; Eisenberg et al., 1997). Additionally, ProSA was used to determine overall model quality based on Z-score evaluation, reflecting the structural plausibility of the predicted models (https://prosa.services.came.sbg.ac.at/prosa.php) (Wiederstein and Sippl, 2007). Only models satisfying established quality criteria across all validation parameters were selected for subsequent molecular docking studies.

#### 2.2.2 Preparation of proteins and ligand for molecular docking, interface characterization and interaction analysis

Molecular docking was performed to evaluate the binding affinity between SAMe and the selected hub proteins using AutoDock (version 1.5.6) (Morris et al., 2009) and AutoDock Vina (Eberhardt et al., 2021). The three-dimensional structure of SAMe was retrieved from the PubChem database (https://pubchem.ncbi.nlm.nih.gov/) (Kim et al., 2021) (CID - 34,756) and converted into PDBQT format using Open Babel following energy minimization and addition of hydrogen atoms (O’Boyle et al., 2011). Crystal structures of experimentally resolved target proteins were obtained from the RCSB Protein Data Bank (https://www.rcsb.org/) (Berman et al., 2000; Zardecki et al., 2016), including TGFB1 (PDB ID - 5VQP), ESR1 (PDB ID - 5ACC), PDGFRA (PDB ID - 5 GR N) and BCL2 (PDB ID - 5FCG). The validated homology models of COL1A2 and LUM were also included in the docking analysis. Prior to docking, protein structures were prepared by removing water molecules, adding polar hydrogens and assigning appropriate atomic charges. Grid boxes were defined to encompass the active or potential binding regions of each protein and the corresponding grid parameters are summarized in Table 1. Molecular docking was then carried out using AutoDock Vina, employing a blind docking approach to allow comprehensive exploration of potential binding sites. The resulting protein-ligand complexes were subsequently visualized and analysed using PyMOL (PyMOL pymol.org, 2026; Yuan et al., 2017) and Discovery Studio (BIOVIA Discovery Studio Dassault Systèmes, 2026) to examine binding conformations and detailed interaction profiling, including the generation of 2D interaction diagrams. The best binding conformations were selected based on minimum binding energy and favourable interaction profiles and were subsequently used for further analysis.

**TABLE 1:** Docking grid parameters for selected hub proteins used in molecular docking analysis of SAMe.

Target protein	UniProt ID	PDB ID	Spacing	Number of points			Centre		
				x	y	z	x	y	z
TGFB1	P01137	5VQP	0.375	126	126	126	86.908	43.587	36.18
ESR1	P03372	5ACC	0.375	126	126	96	12.512	33.891	68.923
PDGFRA	P16234	5 GR N	0.375	126	126	126	−11.417	10.037	−15.096
BCL2	P10415	5FCG	0.375	114	110	92	75.198	181.072	2.905
COL1A2	P08123	SWISS MODEL	0.375	126	126	126	−4.005	57.917	109.171
LUM	P51884	SWISS MODEL	0.375	126	126	126	−31.039	20.775	116.187

The table includes UniProt IDs, PDB IDs, grid dimensions (number of points along x, y, z-axes) and corresponding centre coordinates used to define the ligand-binding region.

#### 2.2.3 Molecular dynamics simulations

To investigate the stability and dynamic behaviour of the protein-ligand complexes at the atomic level, molecular dynamics (MD) simulations were performed using GROMACS (version 2025) (https://www.gromacs.org/Downloads) (Abraham et al., 2015). The best-scoring docked complexes, including TGFB1-SAMe, PDGFRA-SAMe, COL1A2-SAMe and LUM-SAMe, were selected for simulation studies. Topology files for the proteins were generated using the CHARMM27 all-atom force field, while ligand parameters for SAMe were obtained using SWISS Param (Bugnon et al., 2023). Each system was solvated in a cubic simulation box using the TIP3P water model and appropriate terminal groups (NH_3_^+^ and COO^−^) were assigned. Counter ions (Na^+^ or Cl^−^) were added to neutralize the system and maintain electrostatic stability. Energy minimization was carried out using the steepest descent algorithm for 5,000 steps to remove steric clashes and optimize system geometry. This was followed by two equilibration phases: the NVT ensemble (constant number of particles, volume and temperature) and the NPT ensemble (constant number of particles, pressure and temperature). During equilibration, the system temperature was maintained at 300 K using a thermostat, while pressure was stabilized at 1 bar using the Parrinello-Rahman barostat.

Subsequently, production MD simulations were conducted for 100 ns, with trajectory coordinates recorded for every two femtoseconds. Post-simulation analyses were performed to evaluate structural stability and conformational dynamics, including root mean square deviation (RMSD), root mean square fluctuation (RMSF), radius of gyration (Rg), solvent-accessible surface area (SASA) and hydrogen bond interactions. The resulting trajectories were analysed and visualized using Xmgrace software (Turner, 2026. XMGRACE, Version 5.1.19. Center for Coastal and Land-Margin Research; Oregon Graduate Institute of Science and Technology: Beaverton, OR, 2005; Google Search, n.d.).

#### 2.2.4 Binding free energy analysis

To evaluate the thermodynamic stability of the protein-ligand interactions, binding free energy (ΔG) was calculated using the molecular mechanics/generalized Born surface area (MM/GBSA) method (Genheden and Ryde, 2015). This approach estimates the free energy difference between the bound and unbound states of protein-ligand complexes by integrating molecular mechanics energies with solvation contributions. Binding free energy calculations were performed on the equilibrated trajectories derived from MD simulations, considering the final 20 ns of each system, including TGFB1-SAMe, PDGFRA-SAMe, COL1A2-SAMe and LUM-SAMe complexes. The total binding free energy (ΔG_(Total)_) was computed using the following equations:

$${\Delta G}_{\left(Total\right)}={\Delta E}_{\left(MM\right)\;}+{\Delta G}_{\left(solv\right)}-T\Delta S$$

$${\Delta E}_{\left(MM\right)}={\;\Delta E}_{\left(EEL\right)}+{\Delta E}_{\left(vdW\right)}$$

$${\Delta G}_{\left(solv\right)}={\Delta G}_{\left(polar\right)}+{\Delta G}_{\left(nonpolar\right)}$$

where ΔE_(MM)_ represents the molecular mechanics energy, comprising electrostatic (ΔE_(EEL)_) and van der Waals (ΔE_(vdW)_) interactions. ΔG_(solv)_ denotes the solvation free energy, including polar (ΔG_(polar)_) and non-polar (ΔG_(nonpolar)_) components. In the MM/GBSA framework, polar solvation energy was estimated using the Generalized Born (GB) model, while non-polar solvation energy was calculated based on SASA. The entropic contribution (TΔS), reflecting conformational flexibility and disorder, was considered in the overall free energy estimation. This energy decomposition approach enables detailed characterization of the forces governing ligand binding and allows comparative assessment of interaction stability of different complexes. Consequently, MM/GBSA analysis provides valuable mechanistic insight into the binding interactions of the protein-ligand complexes.

## 3 Results

### 3.1 Identification of hub genes

#### 3.1.1 Identification of DEGs and overlapping genes

**FIGURE 1:**
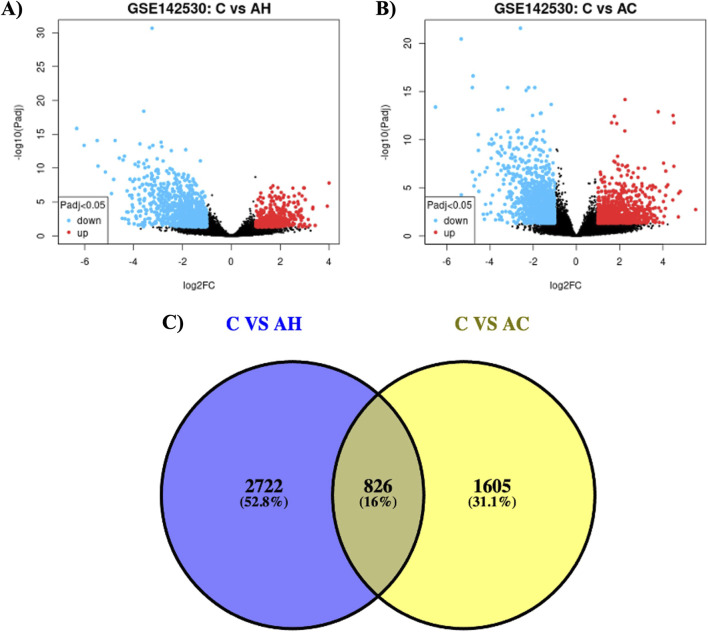
Differential gene expression analysis and identification of overlapping differentially expressed genes (DEGs) between alcoholic hepatitis (AH) and alcoholic cirrhosis (AC) **(A)** Volcano plot illustrating DEGs in the Control (C) vs. AH comparison, displaying log_2_ fold change versus -log_10_ (adjusted *p*-value) **(B)** Volcano plot showing DEGs in the C vs. AC comparison under the same criteria. Upregulated genes are shown in red, while downregulated genes are shown in blue based and statistical significance (adjusted *p* < 0.05) **(C)** Venn diagram representing the overlap between DEGs identified in both comparisons, highlighting 826 commonly dysregulated genes (16%) shared between AH and AC conditions.

Differential gene expression analysis was conducted to characterize transcriptional alterations between control (C) samples and disease conditions, including alcoholic hepatitis (AH) and alcoholic cirrhosis (AC). The distribution of DEGs is illustrated using volcano plots, representing log_2_ fold change versus statistical significance (adjusted *p* < 0.05) (Figures 1A,B). In the C vs. AH comparison, a substantial number of genes exhibited significant differential expression, with a predominance of downregulated transcripts, as indicated by negative log_2_ fold change values. Several genes demonstrated marked transcriptional alterations with high statistical significance, reflected by -log_10_ (p-value) exceeding 20. In contrast, the C vs. AC comparison showed a more balanced distribution of upregulated and downregulated genes, indicating a broader and more complex transcriptional reprogramming. Notably, a greater number of genes in the C vs. AC comparison exhibited higher fold changes and stronger statistical significance relative to the C vs. AH comparison, suggesting enhanced transcriptional dysregulation in the advanced disease stage. To identify conserved molecular signatures, overlapping DEGs between AH and AC were determined using Venn diagram analysis. A total of 2,722 genes (52.8%) were uniquely associated with the C vs. AH comparison, while 1,605 genes (31.1%) were specific to the C vs. AC comparison. Importantly, 826 genes (16%) were commonly dysregulated across both conditions, representing a subset of shared transcriptional alterations (Figure 1C). These common DEGs were prioritized for subsequent functional enrichment and network-based analyses.

#### 3.1.2 Functional enrichment analysis of shared DEGs

**FIGURE 2:**
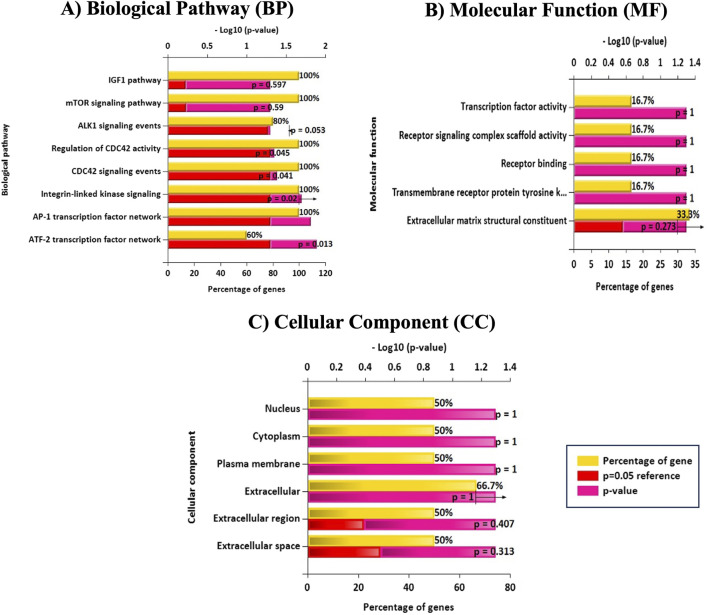
Functional enrichment analysis of overlapping DEGs between AH and AC **(A)** Biological process (BP) **(B)** molecular function (MF) and **(C)** cellular component (CC) categories derived from Gene Ontology (GO) analysis. The bar plots represent enriched functional terms, with gene distribution shown as percentage and statistical significance represented as -log_10_ (p-value).

Functional enrichment analysis was performed to elucidate the biological significance of the shared DEGs between AH and AC using FunRich and KEGG pathway databases. Gene Ontology (GO) analysis revealed significant enrichment of these genes in biological process (BP) associated with intracellular signalling and transcriptional regulation, including integrin-linked kinase signalling, CDC4-related regulatory pathways, ALK1 signalling and ATF-2/AP-1 transcription factor networks (Figure 2A). KEGG pathway analysis further indicated that the shared DEGs were predominantly involved in signalling cascades governing cellular growth, signal transduction and cytoskeletal organization, notably including the IGF1/mTOR signalling pathway. These findings suggest the involvement of coordinated regulatory mechanisms controlling cellular proliferation and metabolic adaptation in disease progression.

Cellular component (CC) analysis demonstrated that a substantial proportion of the shared DEGs were localized to the extracellular space, plasma membrane, cytoplasm and nucleus, indicating their participation in both extracellular signalling and intracellular regulatory processes (Figure 2B). In parallel, molecular function (MF) enrichment highlighted activities related to extracellular matrix structural constituents, receptor tyrosine kinase activity, receptor complex scaffolding and transcription factor binding (Figure 2C). Collectively, these results indicate that the shared DEGs between AH and AC are functionally enriched in interconnected signalling and transcriptional regulatory networks, particularly those governing cell-matrix interactions and receptor-mediated signalling (Table 2).

**TABLE 2:** Functional enrichment analysis of overlapping differentially expressed genes derived from FunRich and KEGG analyses.

GO terms	Percentage of the gene (%)	-log_10_ (p-value)	Fold
Molecular function (MF)			
Extracellular matrix structural constituent	33.3%	0.56	36.5
Transmembrane receptor protein tyrosine kinase activity	16.7%	0.00	54.4
Receptor binding	16.7%	0.00	23.6
Receptor signalling complex scaffold activity	16.7%	0.00	9.5
Transcription factor activity	16.7%	0.00	3.6
Cellular component (CC)			
Extracellular space	50%	0.05	18
Extracellular region	50%	0.39	16.5
Extracellular	50%	0.00	5.3
Plasma membrane	50%	0.00	2.1
Cytoplasm	50%	0.00	1.3
Nucleus	50%	0.00	1.2
Biological process (BP)			
ATF-2 transcription factor network	60%	1.89	64
AP-1 transcription factor network	100%	1.81	10.1
Integrin-linked kinase signalling	100%	1.70	9.6
CDC4 signalling events	100%	1.39	8.3
Regulation of CDC42	100%	1.35	8.2
ALK1signalling events	80%	1.27	15.7
IGF1 pathway mTOR signalling pathway	100%	0.23	4.9

The table summarizes Gene Ontology (GO) categories, including biological process (BP), molecular function (MF) and cellular component (CP), with corresponding gene distribution percentages, statistical significance (-log_10_ p-value) and fold enrichment values.

#### 3.1.3 Protein-protein interaction network construction and hub gene identification

**FIGURE 3:**
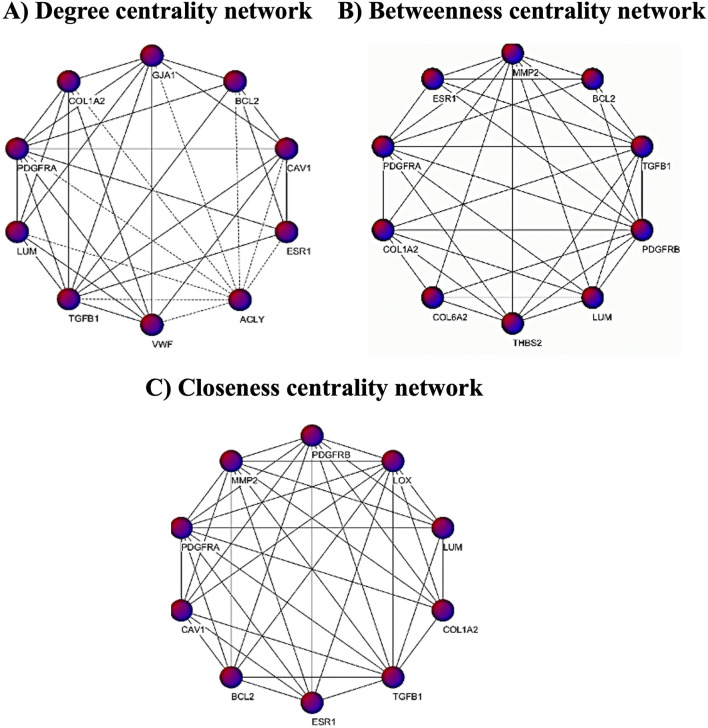
Protein-protein interaction (PPI) network analysis highlighting top-ranked hub genes based on network centrality measures **(A)** Degree centrality network **(B)** betweenness centrality network and **(C)** closeness centrality network, each representing the top 10 ranked genes within the interaction network. Nodes indicate genes, while edges represent functional associations between them.

A protein-protein interaction (PPI) network was constructed to investigate the functional relationships among the shared DEGs identified between AH and AC. The resulting network comprised 549 nodes and 2,245 edges, with an average node degree of 8.18 and an average local clustering coefficient of 0.443. The observed number of interactions was significantly greater than expected (expected edges = 1,024; PPI enrichment *p* < 1.0 × 10^−16^). The resulting network exhibited extensive interconnectivity among the shared DEGs, indicating the presence of coordinated molecular interactions associated with disease progression. Based on degree centrality, TGFB1, COL1A2, ESR1, PDGFRB, LUM, MMP2, PDGFRA, COL6A2, BCL2 and THBS2 emerged as the top-ranking nodes, reflecting high interaction connectivity within the network (Figure 3A). Further analyses using betweenness and closeness centrality consistently identified ESR1, LUM, CAV1, BCL2, PDGFRA, TGFB1 and COL1A2 as key intermediary nodes positioned centrally within the interaction network (Figures 3B,C). Integrative analysis of the topological parameters collectively prioritized TGFB1 (A), ESR1 (B), PDGFRA (C), BCL2 (D), COL1A2 (E) and LUM (F) as the principal hub genes. These genes displayed strong connectivity and central network positioning, suggesting their potential involvement in the shared molecular mechanisms underlying AH and AC progression (Table 3).

**TABLE 3:** Topological ranking of hub genes identified from the protein-protein interaction network using Cytoscape.

Hub genes	Score
Degree centrality	
TGFB1	142
COL1A2	132
ESR1	124
PDGFRB	120
LUM	118
MMP2	114
PDGFRA	114
COL6A2	106
BCL2	104
THBS2	100
Betweenness centrality	
ESR1	33,192.35
CAV1	26,682.46
LUM	16,090.43
BCL2	16,072.4
PDGFRA	13,859.87
GJA1	13,581.54
VWF	12,689.77
TGFB1	11,932.41
COL1A2	11,738.55
ACLY	11,457.05
Closeness centrality	
TGFB1	249.0833
ESR1	245.4833
PDGFRA	241.8333
PDGFRB	241.55
MMP2	240.6333
CAV1	238.65
BCL2	236.7333
COL1A2	234.95
LUM	233.65
LOX	233.6167

The table summarizes the top 10 genes in each category based on degree centrality, betweenness centrality and closeness centrality scores.

### 3.2 Examination of binding affinity, molecular dynamics and binding free energy

#### 3.2.1 Molecular docking analysis

To evaluate the binding interactions between S-adenosyl-L-methionine (SAMe) and the identified hub proteins, molecular docking analysis was performed against TGFB1 (A), ESR1 (B), PDGFRA (C), BCL2 (D), COL1A2 (E) and LUM (F) using AutoDock Vina. Prior to docking, the unavailable protein structures of COL1A2 and LUM were modelled using the SWISS-MODEL server. The generated COL1A2 model (chain A) covered 62.68% of the target sequence (1,130–1,365 aa) with a GMQE score of 0.68, while the LUM model exhibited 89.35% sequence coverage (36–337 aa) with a GMQE score of 0.76, indicating reliable model quality. Structural validation further confirmed the suitability of the predicted models for downstream docking analysis. VERIFY3D analysis showed that 84.96% and 85.43% of residues in COL1A2 and LUM, respectively, achieved compatibility scores ≥0.1. Similarly, ERRAT quality factors were 93.86 for COL1A2 and 77.89 for LUM, indicating acceptable structural reliability. ProSA analysis yielded Z-scores of −6.39 and −5.67 for COL1A2 and LUM, respectively, further supporting the structural stability of the generated models (Table 4).

**TABLE 4:** Structural validation of homology-modelled protein structures of collagen type I alpha 2 chain (COL1A2) and lumican (LUM) using multiple structural assessment computational tools.

Tools/Webservers	Outcomes	Collagen type I alpha 2 chain (COL1A2)		Lumican (LUM)	
		Number of amino acids	Coverage	Number of amino acids	Coverage
ProCheck	Residues in most favoured regions [A,B,L]	176	88.40%	202	74.00%
	Residues in additional allowed regions [a,b,l,p]	19	9.50%	70	25.60%
	Residues in generously allowed regions [∼a,∼b,∼l,∼p]	4	2.00%	1	0.40%
	Residues in disallowed regions	0	0.00%	0	0.00%
	Number of non-glycine and non-proline residues	199	100.00%	273	100.00%
	Number of end-residues (excl. Gly and pro)	2		2	
	Number of glycine residues (shown as triangles)	15		11	
	Number of proline residues	10		16	
	Total number of residues	226		302	
VERIFY 3D	Compatibility of the 3D model		84.96%		85.43%
ERRAT	Overall model quality factor		93.87		77.89
ProSA	z-score		−6.39		−5.67

The table presents the validation methods, corresponding outcomes, number of amino acids analysed and sequence coverage values for each model.

**FIGURE 4:**
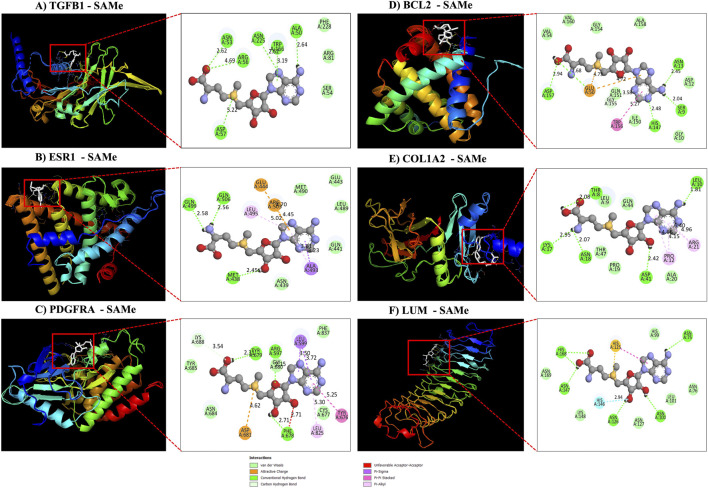
Molecular docking analysis of S-adenosyl-L-methionine (SAMe) with key hub proteins identified from the PPI network **(A)** TGFB1-SAMe **(B)** ESR1-SAMe **(C)** PDGFRA-SAMe **(D)** BCL2-SAMe **(E)** COL1A2-SAMe and **(F)** LUM-SAMe complexes. For each target, both three-dimensional (3D) binding conformations and two-dimensional (2D) interaction maps are presented. The 3D structures illustrate the spatial orientation of SAMe within the binding pocket, while the 2D interaction diagrams depict key amino acid residues involved in ligand binding. Coloured dashed lines represent different types of intermolecular interactions, including hydrogen bonds and hydrophobic contacts, highlighting the molecular basis of ligand-protein stabilization.

Docking analysis revealed differential binding affinities between SAMe and the selected target proteins. Among all complexes, TGFB1 exhibited the strongest binding affinity with a docking score of −7.0 kcal/mol and formed six hydrogen bonds involving key interacting residues ALA50, ASN53, ASP57, TRP166 and ASN225. COL1A2 and LUM also demonstrated favourable binding energies of −6.8 kcal/mol and −6.2 kcal/mol, respectively, with multiple hydrogen bond interactions. Key interacting residues identified in the COL1A2 complex included THR1148, LEU1150, PRO1152, LYS1157, ASN1158, ARG1161 and ASP1181, whereas LUM interactions involved ASN75, ASN100, HIS125, ASN126, HIS146, ASN147 and HIS168. In addition, PDGFRA, ESR1 and BCL2 exhibited binding energies of −6.4, −5.9 and −5.7 kcal/mol, respectively, and formed three to four hydrogen bonds with SAMe (Figures 4A–F). Based on the molecular docking results, TGFB1, COL1A2, PDGFRA and LUM were prioritized for subsequent molecular dynamics (MD) simulations. These protein-ligand complexes exhibited more favourable binding affinities and a greater number of stabilizing hydrogen-bond interactions with SAMe compared with ESR1 and BCL2. In addition, the established involvement of these proteins in extracellular matrix remodelling, TGF-β signalling, collagen assembly and tissue repair further supported their selection for detailed dynamic stability analyses (Table 5).

**TABLE 5:** Binding interaction profile of S-adenosyl-L-methionine (SAMe) with selected target proteins obtained from molecular docking analysis.

Complex	Binding energy (Kcal/mol)	Number of hydrogen bonds	Interactive residues
TGFB1-SAMe	−7	6	ALAA:50, ASNA:53, ARGA:56, ASPA:57, TRPA:166, ASNA:225
ESR1-SAMe	−5.9	3	META:438, GLUA:444, ALAA:493, LEUA:495, GLNA:499, ARGA:503, GLNA:506
PDGFRA-SAMe	−6.4	3	ARGA:597, LEUA:599, TYRA:676, PHEA:678, TYRA:679, ASPA:681, LYSA:688, LEUA:825
BCL2-SAMe	−5.7	4	SERA:9, ASNA:13, GLUA:50, HISA:147, GLYA:155, TRPA:156, ASPA:157
COL1A2-SAMe	−6.8	5	THRA:1,148, LEUA:1,150, PROA:1,152, LYSA:1,157, ASNA:1,158, ARGA:1,161, ASPA:1,181
LUM-SAMe	−6.2	5	ASNA:75, ASNA:100, HISA:125, ASNA:126, HISA:146, ASNA:147, HISA:168

The table summarizes binding energies (kcal/mol), number of hydrogen bonds and key amino acid residues involved in ligand-protein interactions.

#### 3.2.2 Molecular dynamics simulations

**FIGURE 5:**
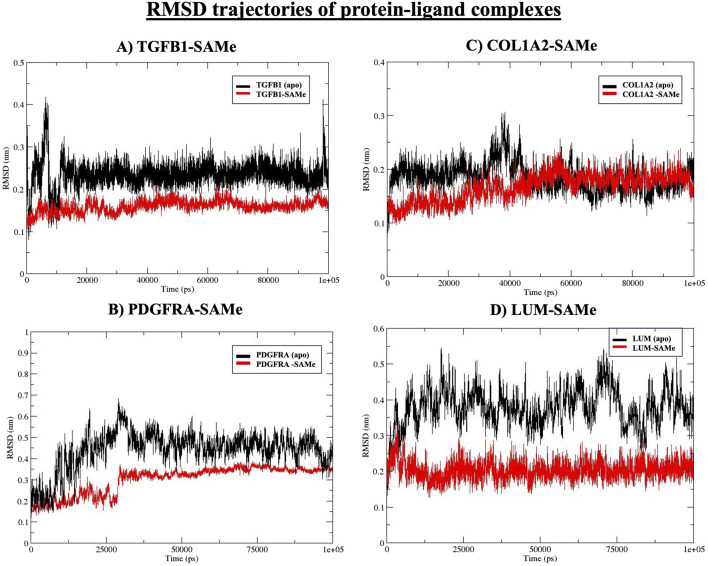
Root mean square deviation (RMSD) trajectories of selected apo proteins and SAMe-bound complexes over 100 ns molecular dynamics simulations. **(A)** TGFB1-SAMe **(B)** PDGFRA-SAMe **(C)** COL1A2-SAMe and **(D)** LUM-SAMe. The black curves represents the apo protein, while the red curves correspond to their respective SAMe-bound complex. RMSD values (nm) are plotted as a function of simulation time (ps), illustrating the conformational stability and equilibration behaviour of each system.

To further evaluate the structural stability and dynamic behaviour of the protein-ligand complexes, 100 ns MD simulations were performed for TGFB1-SAMe, PDGFRA-SAMe, COL1A2-SAMe and LUM-SAMe systems. Comparative analysis of root mean square deviation (RMSD) trajectories demonstrated that all SAMe-bound complexes achieved stable conformational behaviour throughout the simulation period relative to their corresponding apo forms. Among the analyzed systems, the TGFB1-SAMe complex exhibited rapid equilibration within the initial 10 ns and subsequently maintained a highly stable RMSD profile ranging from 0.12 to 0.18 nm, indicating enhanced backbone stability upon ligand binding (Figure 5A). The PDGFRA-SAMe complex displayed comparatively higher RMSD fluctuations (0.15–0.35 nm), likely attributable to the larger structural architecture of PDGFRA; however, the deviations remained stable without major conformational drift (Figure 5B). Similarly, the COL1A2-SAMe complex maintained a stable RMSD range between 0.12 and 0.22 nm following initial equilibration (Figure 5C), whereas the LUM-SAMe complex exhibited moderate but stable fluctuations within 0.15–0.30 nm (Figure 5D). Overall, these findings suggest that SAMe binding contributes to structural stabilization of the target proteins.

**FIGURE 6:**
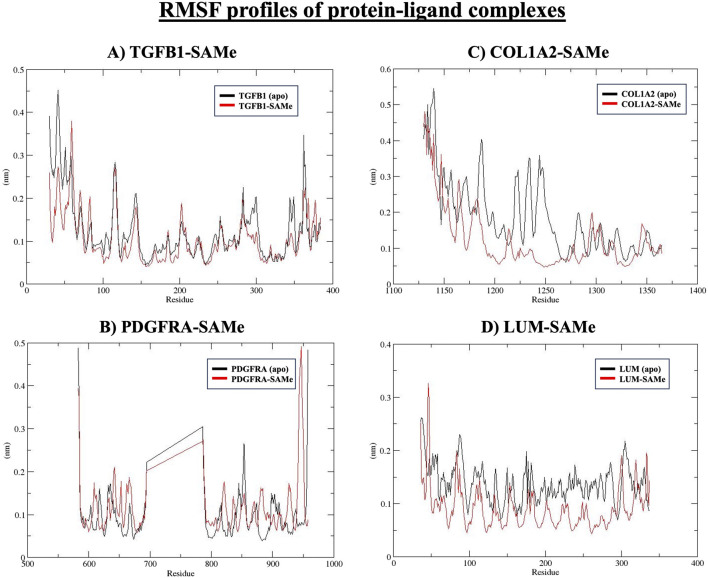
Root mean square fluctuation (RMSF) profiles of selected apo proteins and SAMe-bound complexes over 100 ns molecular dynamics simulations **(A)** TGFB1-SAMe **(B)** PDGFRA-SAMe **(C)** COL1A2-SAMe and **(D)** LUM-SAMe. The black curves represent the apo protein, while the red curves correspond to their respective SAMe-bound complexes. RMSF values (nm) are plotted against residue index, illustrating residue-level flexibility and local conformational fluctuations within each system.

**FIGURE 7:**
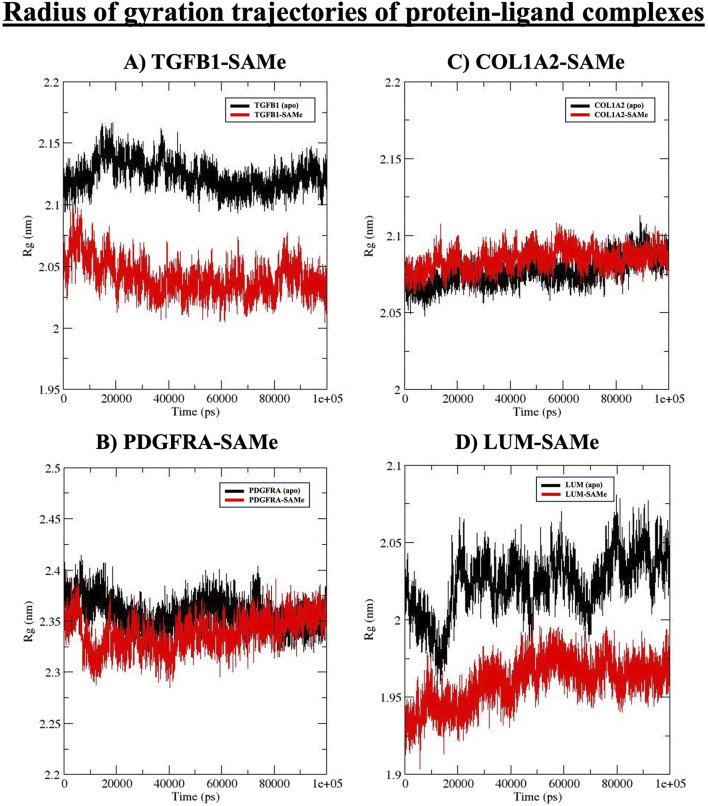
Radius of gyration (Rg) trajectories of selected apo proteins and SAMe-bound complexes over 100 ns molecular dynamics simulations **(A)** TGFB1-SAMe **(B)** PDGFRA-SAMe **(C)** COL1A2-SAMe and **(D)** LUM-SAMe. The black curve represents the apo protein, while the red curve corresponds to the respective SAMe-bound complex. Rg values (nm) are plotted against simulation time (ps), illustrating the compactness and overall structural stability of both apo and ligand-bound systems.

Root mean square fluctuation (RMSF) analysis further demonstrated reduced residue-level flexibility in the ligand-bound systems compared to apo proteins. Most residues fluctuated within the range of 0.05–0.38 nm, with relatively higher fluctuations restricted to terminal and linker regions, while core residues remained structurally stable throughout the simulation (Figures 6A–D). Notably, the binding regions of the COL1A2-SAMe and LUM-SAMe complexes exhibited comparatively lower fluctuations, indicating localized stabilization induced by ligand interaction. The radius of gyration (Rg) analysis supported these observations by demonstrating enhanced compactness in the ligand-bound systems. TGFB1-SAMe maintained a stable Rg profile between 2.01 and 2.10 nm, whereas PDGFRA-SAMe and COL1A2-SAMe exhibited stable ranges of 2.29–2.39 nm and 2.05–2.10 nm, respectively. The LUM-SAMe complex showed slightly lower Rg values (1.95–2.00 nm) compared to its apo form, suggesting reduced structural expansion upon ligand binding (Figures 7A–D).

**FIGURE 8:**
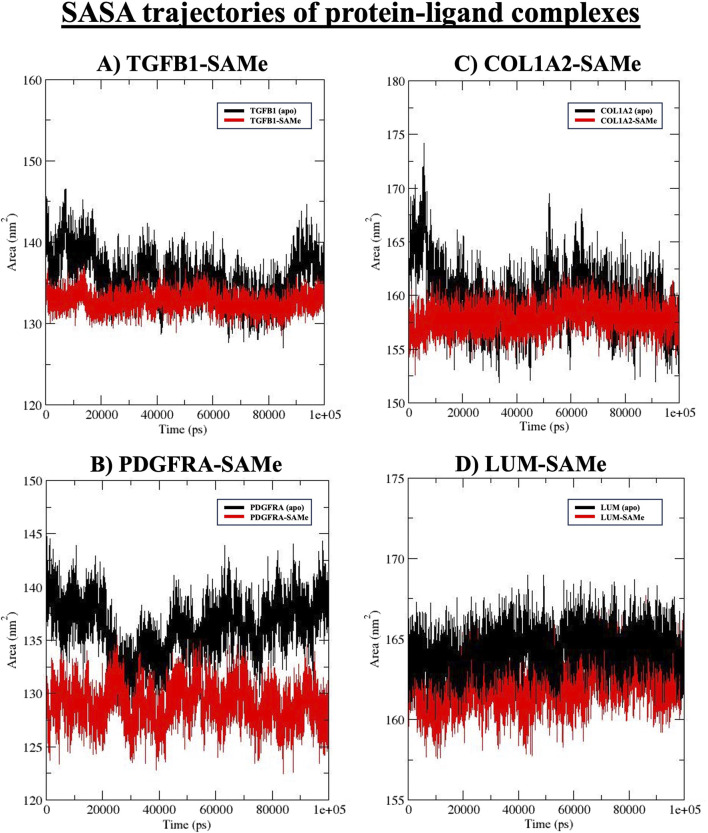
Solvent accessible surface area (SASA) trajectories selected apo proteins and SAMe-bound complexes over 100 ns molecular dynamics simulations **(A)** TGFB1-SAMe **(B)** PDGFRA-SAMe **(C)** COL1A2-SAMe and **(D)** LUM-SAMe. The black curve represents the apo protein, while the red curve corresponds to the respective SAMe-bound complex. SASA values (nm^2^) are plotted against simulation time (ps), illustrating changes in solvent exposure and surface accessibility of both apo and ligand-bound systems.

**FIGURE 9:**
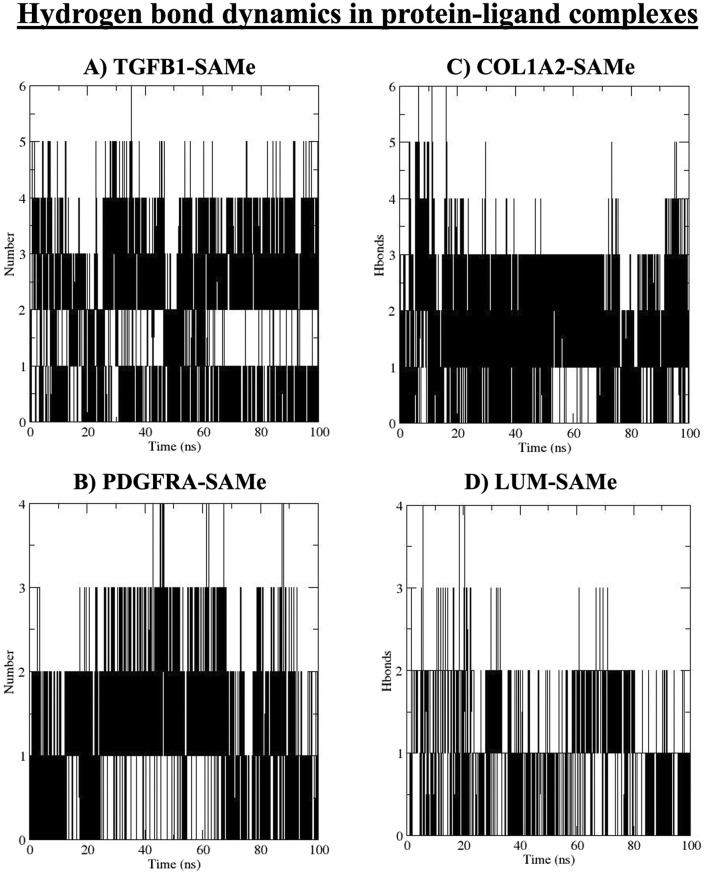
Hydrogen bond dynamics of SAMe-protein complexes over 100 ns molecular dynamics simulations **(A)** TGFB1-SAMe **(B)** PDGFRA-SAMe **(C)** COL1A2-SAMe and **(D)** LUM-SAMe. The number of hydrogen bonds is plotted against simulation time (ns), illustrating the stability and persistence of intermolecular interactions within each protein-ligand complex.

Similarly, solvent accessible surface area (SASA) analysis revealed decreased solvent exposure in all complexes during the simulation period. TGFB1-SAMe and COL1A2-SAMe maintained stable SASA values within 130–135 nm^2^ and 155–160 nm^2^, respectively, while PDGFRA-SAMe and LUM-SAMe exhibited values ranging from 125 to 135 nm^2^ and 157–162 nm^2^ (Figures 8A–D). The reduced SASA profiles indicate the formation of more compact and energetically stable conformations following ligand association. Hydrogen bond analysis further highlighted the stability of the complexes, with TGFB1-SAMe and COL1A2-SAMe consistently maintaining five to six intermolecular hydrogen bonds throughout the simulation. In contrast, PDGFRA-SAMe and LUM-SAMe formed three to four hydrogen bonds, indicating moderate yet stable ligand interactions within the binding pockets (Figures 9A–D). Collectively, the MD simulation results demonstrate that SAMe binding enhances conformational stability, reduces residue fluctuations, promotes structural compactness and maintains stable intermolecular interactions across multiple hub proteins (Table 6).

**TABLE 6:** Comparative molecular dynamics simulation analysis of apo proteins and SAMe-bound complexes.

System	RMSD range (nm)	RMSF range (nm)	Rog (nm)	SASA (nm^2^)	Number of hydrogen bonds
TGFB1 (apo)	∼0.20–0.35	∼0.15–0.45	∼2.10–2.17	∼135–146	0
TGFB1-SAMe	∼0.12–0.18	∼0.12–0.38	∼2.01–2.10	∼130–135	5–6
PDGFRA (apo)	∼0.30–0.65	∼0.14–0.48	∼2.35–2.42	∼130–145	0
PDGFRA-SAMe	∼0.15–0.35	∼0.12–0.28	∼2.29–2.39	∼125–135	3–4
COL1A2 (apo)	∼0.15–0.28	∼0.20–0.54	∼2.08–2.11	∼155–172	0
COL1A2-SAMe	∼0.12–0.22	∼0.10–0.45	∼2.05–2.10	∼155–160	5–6
LUM (apo)	∼0.30–0.53	∼0.10–0.26	∼1.96–2.08	∼162–168	0
LUM-SAMe	∼0.15–0.30	∼0.08–0.25	∼1.95–2.00	∼157–162	3–4

The table summarizes RMSD range (nm), RMSF range (nm), Rg (nm), SASA (nm^2^) and number of hydrogen bonds, highlighting differences in structural stability and interaction dynamics.

#### 3.2.3 Binding free energy calculations

**FIGURE 10:**
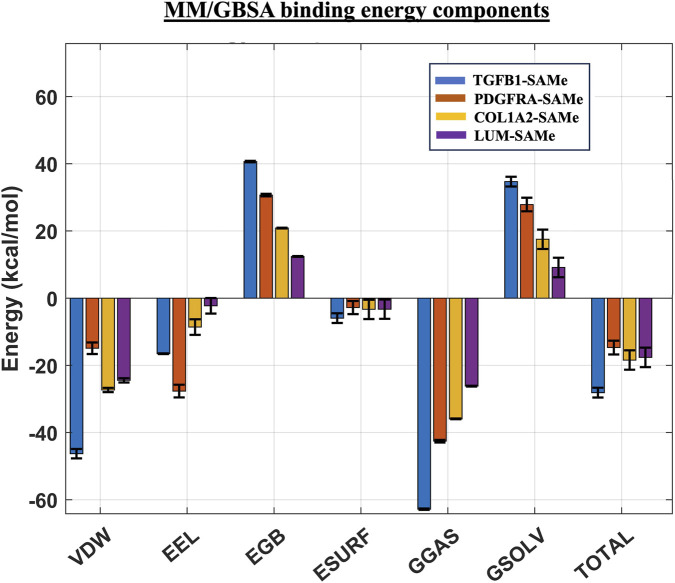
MM/GBSA-based binding free energy decomposition of selected SAMe-protein complexes. The bar graph illustrates individual energy components, including van der Waals (ΔVDWAALS), electrostatic (ΔEEL), polar solvation (ΔEGB), non-polar solvation (ΔESURF), gas-phase binding energy (ΔGGAS), solvation free energy (ΔGSOLV) and total binding free energy (ΔTOTAL), with energy terms represented on the x-axis and their corresponding energy contributions (kcal/mol) on the y-axis. Different colours denote individual complexes: TGFB1-SAMe (blue), PDGFRA-SAMe (orange), COL1A2-SAMe (yellow) and LUM-SAMe (purple), enabling comparative evaluation of interaction energetics across targets.

The thermodynamic stability of the protein-ligand complexes was further evaluated using MM/GBSA binding free energy calculations by analysing the bound and unbound states of the receptor-ligand systems. Across all complexes, van der Waals interaction energies ranged from −24.46 to −46.28 kcal/mol, indicating favourable hydrophobic interactions within the binding pockets that contributed substantially to ligand stabilization. Among the analysed systems, the TGFB1-SAMe complex exhibited the strongest electrostatic interaction energy (−16.53 kcal/mol), suggesting enhanced electrostatic stabilization relative to the other complexes. The total gas-phase energy, which represents the combined contribution of van der Waals and electrostatic interactions, was also lowest for the TGFB1-SAMe complex (−62.82 kcal/mol), indicating a highly favourable interaction profile. In addition, non-polar solvation energy values for all complexes ranged between −3.27 and −5.95 kcal/mol, further supporting the energetic stability of the ligand-bound systems. The consistently negative solvation and interaction energies indicate favourable binding energetics and stable receptor–ligand associations (Figure 10).

Comparative analysis of total binding free energy revealed that SAMe exhibited the strongest binding affinity toward TGFB1, with a ΔG value of −28.12 kcal/mol. This was followed by COL1A2 (−18.41 kcal/mol), LUM (−17.62 kcal/mol) and PDGFRA (−15.48 kcal/mol). The markedly lower binding free energy observed for the TGFB1-SAMe complex suggests enhanced thermodynamic stability and stronger interaction potential compared to the other target proteins. Overall, the MM/GBSA analysis corroborated the molecular docking and molecular dynamics findings by demonstrating favourable energetic profiles and stable intermolecular interactions for the SAMe-bound complexes (Table 7). Among the evaluated targets, TGFB1 consistently exhibited the most stable interaction with SAMe.

**TABLE 7:** Binding free energy analysis of SAMe-protein complexes calculated using the MM/GBSA method.

Complex	ΔVDWAALS (Kcal/mol)	ΔEEL (Kcal/mol)	ΔEGB (Kcal/mol)	ΔESURF (Kcal/mol)	ΔGGAS (Kcal/mol)	ΔGSOLV (Kcal/mol)	ΔTOTAL (Kcal/mol)
TGFB1-SAMe	−46.28	−16.53	40.65	−5.95	−62.82	34.69	−28.12
PDGFRA-SAMe	−33.04	−8.88	29.7	−3.27	−41.91	26.43	−15.48
COL1A2-SAMe	−27.33	−8.61	20.86	−3.34	−35.94	17.52	−18.41
LUM-SAMe	−24.46	−2.28	12.41	−3.28	−26.75	9.13	−17.62

The table presents energy components including van der Waals (ΔVDWAALS), electrostatic (ΔEEL), polar solvation (ΔEGB), non-polar solvation (ΔESURF), gas-phase binding energy (ΔGGAS), solvation free energy (ΔGSOLV) and total binding free energy (ΔTOTAL).

## 4 Discussion

Alcohol-associated liver disease (ALD) represents a progressive spectrum of hepatic injury driven by interconnected processes including metabolic dysfunction, chronic inflammation, oxidative stress and fibrotic remodelling (Mackowiak et al., 2024; Mandrekar and Mandal, 2024). Despite advances in supportive care and symptom management, the molecular mechanisms underlying the transition from alcoholic hepatitis (AH) to alcoholic cirrhosis (AC) remain incompletely understood. Identifying conserved molecular alterations across these disease stages may therefore provide important insights into the biological processes associated with disease progression. Recent advances in systems biology have demonstrated that shared hub genes across related disease states frequently function as central regulators of disease progression and represent promising candidates for targeted intervention (Hu et al., 2016; Zanello et al., 2023; Ahmmed et al., 2024; Pradeep et al., 2025). Notably, an integrative transcriptomic analysis across multiple cancers have identified conserved hub genes with diagnostic, prognostic and therapeutic significance, while similar computational approaches in metabolic disorders, such as Type 1 diabetes, have revealed key progression-associated molecular targets (Prashanth et al., 2021; Alam et al., 2025). These findings collectively support the premise that identifying shared molecular drivers across progressive disease states provides a rational framework for mechanism-driven therapeutic development. In this context, S-adenosyl-L-methionine (SAMe), a biologically active methyl donor, plays a critical role in maintaining hepatic redox balance, glutathione homeostasis and methylation capacity and has been increasingly recognized for its potential to modulate pathogenic pathways in alcohol-induced tissue injury (Lieber et al., 1990; Cederbaum, 2010; Nandagopal et al., 2026b). Building upon these observations, the present study employed an integrative systems biology framework to identify shared molecular determinants underlying AH and AC and to evaluate their potential modulation by SAMe. By combining transcriptomic profiling, network-based target prioritization and structure-based molecular validation, this study provides a comprehensive systems-level perspective on conserved molecular mechanisms associated with ALD progression and their therapeutic tractability.

Differential gene expression analysis revealed pronounced transcriptional dysregulation in both AH and AC, reflecting the extensive molecular perturbations associated with alcohol-induced liver injury. The predominance of downregulated transcripts in AH may reflect acute hepatocellular dysfunction, impaired metabolic activity and early inflammatory stress responses. In contrast, the more balanced distribution of upregulated and downregulated genes in AC suggests a complex and relatively stabilized transcriptional state, consistent with chronic disease adaptation characterized by sustained inflammation, extracellular matrix (ECM) remodeling and fibrogenesis. This shift in transcriptional dynamics across disease stages aligns with prior transcriptomic studies demonstrating that advanced fibrotic conditions exhibit more persistent and amplified gene expression changes compared to early inflammatory states (Ramachandran et al., 2019). Importantly, the identification of 826 shared differentially expressed genes (DEGs) between AH and AC highlights a conserved molecular signature that likely represents core regulatory drivers of ALD progression rather than stage-specific alterations. Similar cross-condition analyses have shown that shared DEGs often correspond to fundamental pathogenic pathways and can serve as robust candidates for therapeutic targeting (Prashanth et al., 2021; Ahmmed et al., 2024; Pradeep et al., 2025). Although the identified shared DEGs may represent conserved molecular mechanisms associated with ALD progression, alternative biological interpretations should also be considered. The observed overlap may reflect common responses to chronic hepatic injury, inflammation, fibrogenesis, extracellular matrix remodelling, or tissue repair processes that are present in both AH and AC. Furthermore, changes in gene expression may arise from alterations in cellular composition within diseased liver tissue rather than direct pathogenic drivers.

Functional enrichment analysis of the shared DEGs further elucidated their involvement in critical biological process and signalling pathways underlying ALD progression. Enrichment in pathways such as integrin-linked kinase signalling, ALK1 signalling and AP-1/ATF-2 transcriptional networks highlights the activation of coordinated signalling cascades that regulate cellular stress responses, inflammation and fibrogenesis. This is consistent with evidence demonstrating that integrin-mediated signalling and transcription factor networks play pivotal roles in hepatic stellate cell activation and fibrosis development (Henderson et al., 2013). Additionally, the identification of IGF1/mTOR signalling pathways underscores the contribution of metabolic and growth-regulatory mechanisms in chronic liver disease, given the established role of mTOR signalling in hepatic metabolism, cell survival and fibrotic progression (Saxton and Sabatini, 2017). Subcellular localization patterns, spanning the extracellular space, plasma membrane, cytoplasm and nucleus, further emphasize the dual involvement of these genes in extracellular communication and intracellular regulation, particularly in mediating cell-matrix interactions and receptor-driven signalling. Correspondingly, enrichment of molecular functions such as ECM structural constituents and receptor tyrosine kinase activity reinforces their role in shaping the hepatic microenvironment and propagating disease-relevant signalling. Collectively, these findings indicate that the shared DEGs are associated with interconnected signalling and regulatory networks relevant to ALD and provide a biological context for the subsequent prioritization of hub genes through network analysis.

Protein-protein interaction (PPI) network analysis of the shared DEGs provided important insights into the molecular architecture underlying the progression from AH to AC. By integrating multiple network topological measures, including degree, betweenness and closeness centrality, highly interconnected genes within the disease network were initially prioritized. Comparison of the top-ranked genes across these three parameters identified TGFB1, COL1A2, ESR1, PDGFRA, LUM and BCL2 as common hub candidates, supporting their robust network significance. Their prioritization for downstream structural analyses was further supported by significant differential expression patterns, enrichment within pathways associated with inflammation, extracellular matrix remodeling, fibrogenesis and cell survival, as well as experimental evidence implicating these genes in ALD and related hepatic disorders. The prominence of TGFB1 and COL1A2 underscores the significance of profibrotic signalling and collagen deposition in ALD progression, consistent with the established role of TGF-β signalling as a master regulator of hepatic fibrosis (Fabregat et al., 2016). Likewise, PDGFRA and LUM point to the involvement of growth factor signalling and ECM organization in hepatic stellate cell activation and tissue remodeling, processes critical for cirrhotic transformation (Kinnman et al., 2000; Baiocchini et al., 2016). The identification of ESR1 and BCL2 further suggests contributions from hormone-mediated signalling and apoptosis regulation in maintaining cellular homeostasis under chronic liver injury (Villa et al., 2002; Czabotar et al., 2014). Importantly, nodes identified through centrality measures, including TGFB1, ESR1 and PDGFRA, may function as central regulatory nodes that coordinate interactions among multiple pathways implicated in ALD progression. Such network-central genes are increasingly recognized as attractive therapeutic targets due to their capacity to regulate multiple downstream pathways (Barabási et al., 2011). Collectively, these findings suggest that the identified hub genes represent important molecular convergence points within the disease network and warrant further investigation to elucidate their functional roles and potential relevance in ALD progression.

Prior to molecular docking, structural validation of the modelled proteins, including COL1A2 and LUM, confirmed their suitability for downstream analyses, thereby ensuring the reliability of the predicted binding interactions. Although the COL1A2 model covered approximately 63% of the full-length protein sequence, the modelled region encompassed the functionally important C-terminal fibrillar collagen NC1 domain involved in collagen assembly and extracellular matrix organization, providing a biologically relevant framework for further interaction analysis (Devos et al., 2023). Among the evaluated targets, TGFB1 exhibited the highest binding affinity with SAMe, supported by multiple hydrogen bond interactions, indicative of a stable and energetically favorable complex. This observation is particularly relevant given the well-established role of TGF-β signaling in hepatic fibrogenesis, where persistent activation of TGFB1 promotes hepatic stellate cell activation, extracellular matrix deposition and fibrosis progression. Previous studies have also reported antifibrotic effects of SAMe in experimental liver injury models, including the modulation of pathways associated with TGFB1 signalling (Karaa et al., 2008; Fabregat et al., 2016; Wang et al., 2019). Consistent with this, favorable binding interactions with COL1A2 and LUM further suggest the potential of SAMe to influence ECM organization and collagen dynamics, both of which are key determinants of cirrhotic remodeling (Nieto and Cederbaum, 2005). Additionally, the observed interactions of SAMe with PDGFRA, ESR1 and BCL2, albeit with comparatively moderate binding affinities, indicate its capacity to engage multiple signalling axes, including growth factor signalling, hormone-mediated regulation and apoptosis pathways. To further assess the biological relevance of the predicted docking poses, the interacting residues were mapped to known functional domains and PPI interfaces of the target proteins. In TGFB1, key SAMe-interacting residues, including ARG56 and ASN225, were located within regions associated with latent complex activation and TGF-β signaling (Daly et al., 2022; Kannan and Mohan, 2025). Similarly, several PDGFRA interacting residues, including LEU599, PHE678 and LEU825, overlapped with functionally important regions encompassing the ATP-binding and inhibitor-recognition pockets (Yang et al., 2023). In LUM, SAMe-binding residues were localized within the leucine-rich repeat domain, a region implicated in collagen binding and fibrillogenesis (Choi et al., 2023). Likewise, the interacting residues identified in COL1A2 were situated within the conserved C-terminal NC1 domain, which plays a critical role in procollagen assembly and extracellular matrix organization (Kamel, 2025). Collectively, the localization of SAMe-binding residues within functionally relevant domains supports the biological plausibility of the predicted interactions and suggests that SAMe may influence signaling and extracellular matrix remodeling processes associated with ALD. Previous studies have demonstrated that SAMe supplementation enhances hepatic glutathione levels and mitigates oxidative stress-mediated liver injury, thereby indirectly influencing pathways associated with fibrosis and cell survival (Lieber, 2002b; Nandagopal et al., 2026b). The present findings complement these observations by providing a structural perspective on potential interactions between SAMe and key regulatory proteins implicated in ALD. Together, these results suggest the possibility that the biological effects of SAMe may extend beyond its established metabolic functions to include interactions with disease-associated molecular targets; however, further experimental validation is required to determine the functional significance of these predicted interactions.

Molecular dynamics simulations provided additional support for the stability of the predicted SAMe-protein complexes under the simulated conditions. The stabilization of RMSD trajectories across all complexes, particularly the rapid equilibration and low deviation observed in the TGFB1-SAMe system, indicates that ligand binding enhances backbone stability and preserves conformational integrity. Although PDGFRA exhibited comparatively higher RMSD values, these remained stable over time, likely reflecting its larger structural architecture rather than instability. In contrast, COL1A2 and LUM demonstrated sustained stability following initial equilibration, suggesting favorable ligand accommodation within their binding domains. At the residue level, reduced RMSF values in SAMe-bound complexes, particularly within functionally relevant regions, indicate restricted local flexibility and enhanced structural stabilization upon ligand binding, consistent with effective protein-ligand engagement. Consistent with these observations, Rg and SASA values revealed increased compactness and reduced solvent exposure upon ligand binding, further supporting the formation of stable protein-ligand conformations. Moreover, the persistence of hydrogen bonds, particularly in TGFB1 and COL1A2 complexes, underscores the contribution of intermolecular interactions to overall system stability. These stabilization patterns align with previous findings that effective ligand binding is associated with reduced conformational fluctuations and enhanced structural compactness (Hollingsworth and Dror, 2018). Collectively, these findings provide additional computational evidence supporting the potential interaction of SAMe with multiple proteins implicated in ALD. However, the biological significance of these interactions remains to be established through experimental validation.

Additionally, MM/GBSA-based binding free energy analysis provided quantitative validation of the interaction strength and thermodynamic stability of SAMe with the identified hub proteins. Across all complexes, favorable van der Waals contributions predominated, indicating that hydrophobic interactions play a key role in stabilizing ligand binding within protein pockets (Varma et al., 2010). In addition, the comparatively strong electrostatic contribution observed in the TGFB1-SAMe complex highlights the importance of polar interactions in enhancing binding specificity and stability (Wong et al., 2013). Consistent with these observations, the TGFB1-SAMe complex exhibited the most favorable total binding free energy and lowest gas-phase energy, underscoring its superior interaction stability relative to other targets and reinforcing its role as a central regulatory node in ALD progression. Favorable binding energies were also observed for COL1A2, LUM and PDGFRA, suggesting that SAMe may interact with structurally and functionally distinct targets with varying degrees of binding affinity. The contribution of non-polar solvation energy across all complexes further supports the formation of stable ligand-protein interactions, consistent with the reduced solvent exposure and increased compactness observed in the MD simulations (Genheden and Ryde, 2015). These results indicate that both hydrophobic and electrostatic interactions play an important role in stabilizing SAMe binding (Ferreira De Freitas and Schapira, 2017; Zhou and Pang, 2018). Such interaction patterns are commonly associated with stable and biologically relevant ligand-target complexes in computational studies (Genheden and Ryde, 2015). Overall, these findings provide strong thermodynamic support that SAMe can form stable interactions with key regulatory proteins, particularly TGFB1, suggesting its potential to modulate important molecular drivers involved in the progression from AH to AC.

## 5 Limitations and future perspectives

Despite providing a comprehensive systems-level analysis, this study has few limitations that should be acknowledged. First, the findings are derived from a single publicly available transcriptomic dataset selected because it contained both AH and AC samples along with appropriate controls and sufficient sample representation. Nevertheless, reliance on a single cohort may limit the generalizability of the findings and validation using independent transcriptomic datasets would strengthen the robustness and reproducibility of the identified molecular signatures. Since, the entire analysis are based on computational approaches they tend to be influenced by cohort composition, sample heterogeneity and experimental variability. The prioritization of shared DEGs assumes that genes commonly dysregulated in AH and AC are associated with conserved disease mechanisms; however, shared expression patterns alone do not establish causality and may also reflect common pathological responses, tissue remodeling, or changes in cellular composition. Differential expression and network-based analyses identify key regulatory genes based on statistical and topological associations but do not establish causal relationships. Likewise molecular docking, MD simulations and MM/GBSA analyses only provide insights into the feasibility and stability of protein-ligand interactions but do not demonstrate biological activity or therapeutic efficacy. Consequently, the predicted SAMe-target interactions should be regarded as hypothesis-generating. Although MD simulations supported the stability of the selected complexes, only a single 100 ns trajectory was performed for each system; therefore, future studies incorporating replicate simulations would provide additional confidence in these findings. Finally, the identified hub genes and predicted SAMe-target interactions were not experimentally validated. Future studies integrating independent transcriptomic cohorts, multi-omics datasets and functional *in vitro* and *in vivo* investigations will be important for confirming the biological relevance and translational potential of the proposed mechanisms.

## 6 Conclusion

In summary, the present study employed an integrative systems biology and structure-based computational framework to elucidate conserved molecular mechanisms associated with the progression from AH to AC. The identification of 826 shared differentially expressed genes revealed a common transcriptional signature associated with disease continuity, while network-based analyses prioritized TGFB1, COL1A2, ESR1, PDGFRA, LUM and BCL2 as biologically relevant hub genes linked to inflammation, extracellular matrix remodeling, fibrogenesis and cell survival pathways. These findings provide insights into molecular processes that may contribute to the progression of ALD. Structure-based analyses identified favorable predicted interactions between SAMe and several prioritized hub proteins, with TGFB1 exhibiting the strongest binding affinity among the evaluated targets. Molecular dynamics simulations and binding free energy analyses further supported the relative stability of the selected protein-ligand complexes under the simulated conditions. Collectively, these findings suggest that SAMe may interact with multiple molecular targets implicated in alcohol-associated liver disease and provide a computational framework for exploring its potential biological relevance. However, the findings of this study should be considered hypothesis-generating and further experimental validation, independent transcriptomic analyses and preclinical investigations are required to determine the functional significance and therapeutic relevance of the proposed interactions.

## Data Availability

The datasets presented in this study can be found in online repositories. The names of the repository/repositories and accession number(s) can be found in the article/supplementary material.
